# 

**Published:** 1881-08-20

**Authors:** 


### TABLE OF CONTENTS.

ORIGINAL AND SELECTED ARTICLES.

Conversations upon the Physical and Mental Hygienė of Girlhood, by T. S. Powell, M.D. Atlanta, 281. Treatment of Pneumonia, by T. B. Greenley, M.D., of Ky, 289. Emphysema and Bronchial Dilatation, by Jas. H. Low, M.D., of New York, 291. Some Points in the Treatment of Hemorrhoids, by William R. D. Blackwood, M. D., of Penn., 293. Value of Bark in Substance, by William A. Dayton, M.D., 296. Ready Method of Preparing Fomentations, 297.

ABSTRACTS AND GLEANINGS.

Post Partum Hemorrhage, 298. The Summer Dysentery of Children, 299. The Easy Administration of Medicine, 301. A New Anæsthetic; Hemorrhage During Labor, 302. Thumb-Sucking; Phosphide of Zinc in Locomotor Ataxy, 303. Adherent Placenta; Enlarged Tonsils, 304. Epigastric Pressure in Obstinate Hiccough; The Bacteria Fallacy Illustrated; To Pass the Esophageal Tube, 305. Iodide of Potassium in Cardiac Dyspnœa; The Land Origin of the Yellow Fever; Chloral in Dysentery, 306. Abortive Treatment of Small Pox; Infantile Diarrhœa; Lister's Antiseptic Treatment in Surgical Wounds, 307. Double Irrigation and Injection Tube; To Terminate the Chloroform Narcosis; A Prominent Physician, 308. Rhus Aromatica; Jamaica Dogwood; Aconite in Remittent Fevers, 309. Diphtheria: Treatment of Cholera Infantum; Cure of Goiter by Fluoric Acid; Radical Cure of Rupture; Ice to the Abdomen in Typhoid Fever, 310.

SCIENTIFIC ITEMS.

Microscopic Examination of Blood; Transatlantic Cables; Providence of Bees, 311. Electrical Science; Bronzing Liquid; To Remove Rusted Bolts, 312.

PRACTICAL NOTES AND FORMULÆ.

Sassafras Oil as a Remedy for Rhus-Poisoning; How to Cover the Odor of Iodoform; Treatment of Hay-Fever; Dysentery and Diarrhœa, 313. For Acute Dysentery; For Dysenteric Diarrhœa, 314. Sulphate of Zinc as a Caustic; Headache of Dyspepsia; Digitalis; Stramonium, 315. Improved Chalk Mixture; Cholera Infantum; Gelseminum; Bichromate of Potash; Chlorate Potassa; Belladonna in Dysentery; Primary Cancer of the Pancreas, 316.

EDITORIAL AND MISCELLANEOUS.

Apology; The Illustrated News; Three Colleges, etc.; Mellin's Food; John B. Lillard & Co.; Piratical Medical Journals, 317; Letter from New York, 318. The International Medical Congress; Book Notices, 319. Receipts; Special Notices, 320.

